# Modulating Memory Performance in Healthy Subjects with Transcranial Direct Current Stimulation Over the Right Dorsolateral Prefrontal Cortex

**DOI:** 10.1371/journal.pone.0144838

**Published:** 2015-12-17

**Authors:** Daniela Smirni, Patrizia Turriziani, Giuseppa Renata Mangano, Lisa Cipolotti, Massimiliano Oliveri

**Affiliations:** 1 Dipartimento di Scienze Psicologiche, Pedagogiche e della Formazione, Università degli Studi di Palermo, Palermo, Italy; 2 NeuroTeam Life and Science, Palermo, Italy; 3 Department of Neuropsychology, National Hospital for Neurology and Neurosurgery, Queen Square, London, United Kingdom; 4 IRCCS Fondazione “SantaLucia”, Roma, Italy; University of Montreal, CANADA

## Abstract

**Objective:**

The role of the Dorsolateral Prefrontal Cortex (DLPFC) in recognition memory has been well documented in lesion, neuroimaging and repetitive Transcranial Magnetic Stimulation (rTMS) studies. The aim of the present study was to investigate the effects of transcranial Direct Current Stimulation (tDCS) over the left and the right DLPFC during the delay interval of a non-verbal recognition memory task.

**Method:**

36 right-handed young healthy subjects participated in the study. The experimental task was an Italian version of Recognition Memory Test for unknown faces. Study included two experiments: in a first experiment, each subject underwent one session of sham tDCS and one session of left or right cathodal tDCS; in a second experiment each subject underwent one session of sham tDCS and one session of left or right anodal tDCS.

**Results:**

Cathodal tDCS over the right DLPFC significantly improved non verbal recognition memory performance, while cathodal tDCS over the left DLPFC had no effect. Anodal tDCS of both the left and right DLPFC did not modify non verbal recognition memory performance.

**Conclusion:**

Complementing the majority of previous studies, reporting long term memory facilitations following left prefrontal anodal tDCS, the present findings show that cathodal tDCS of the right DLPFC can also improve recognition memory in healthy subjects.

## Introduction

Non-pharmacological interventions such as non-invasive brain stimulation techniques for memory difficulties have gained much attention in recent years [1–3]. In particular, transcranial direct current stimulation (tDCS) proved to be an effective method to facilitate memory capacities in young subjects [4–10] as well as in patients with Alzheimer’s disease [4,11–13]. Prefrontal cortex has been a target site for most brain tDCS studies focusing on memory improvement (e.g. [14–16]). Indeed, the role of the prefrontal cortex in recognition memory has been well documented (e.g. [17,18]) and a large body of research explored differences in hemispheric contribution to memory function of dorsolateral prefrontal cortex (DLPFC). Lesion (e.g. [19]), neuroimaging (e.g. [20, 21]), and TMS (e.g. [22–24]) studies have reported material specific laterality effects in the activation of this region during recognition memory, showing that the left and right prefrontal cortex are differentially recruited, respectively, for verbal and non-verbal stimuli. Other studies reported that hemispheric differences in activation of DLPFC are more related to processes of encoding and retrieval [25–30]. The HERA model—Hemispheric Encoding Retrieval Asymmetry (e.g. [31–32]) suggests that the left PFC plays a crucial role in encoding, whereas right PFC is more necessary for retrieval (but see [33]).

Turriziani et al., [24] investigated the effects of repetitive transcranial magnetic stimulation (rTMS) over the left and right DLPFC before retrieval in verbal and non-verbal recognition memory tests. They used inhibitory and excitatory rTMS paradigms in healthy controls and in Mild Cognitive Impairment (MCI) patients. Inhibitory rTMS-trains of the right DLPFC improved recognition memory performance in healthy controls for both verbal and non-verbal memoranda. rTMS-inhibition of the left DLPFC had no effect in the recognition memory performance. Excitatory rTMS trains using intermittent theta burst stimulation (iTBS) over the right DLPFC impaired accuracy on the non-verbal recognition memory task. In contrast, left iTBS had no effect on recognition memory.

RTMS-inhibition of the right DLPFC selectively improved the recognition memory performance of MCI patients with memory deficits.

These findings suggest that inhibition of a brain circuit centered on the right DLPFC may improve long-term memory, probably modulating a domain-general memory retrieval process [28–29] based on DLPFC-mediated inhibition of subcortical and posterior cortical regions to implement executive control [34–35] According to previous neuromodulation studies [36], the right DLPFC is dominant for this form of inhibitory control.

To deeply investigate the role of DLPFC inhibition vs. excitation on modulation of memory performance, in the present study we explored the effects of anodal vs. cathodal tDCS over the right and left DLPFC on recognition memory of non-verbal stimuli. Non-verbal stimuli were used to emphasize lateralized activation of DLPFC in the retrieval phase [23]. The use of tDCS follows-up and complements previous TMS studies because it can better disentangle the behavioral effects of inhibition vs. excitation of DLPFC. In fact, cathodal and anodal tDCS have the property to map inhibitory and excitatory circuits respectively, thus making a more clear-cut distinction between inhibitory and excitatory processes as compared with rTMS. tDCS was applied during the retention phase, before retrieval, to reproduce the procedure of previous studies reporting memory facilitations following rTMS of the right DLPFC [23–24].

If inhibition of the right DLPFC is indeed a neurophysiological mechanism associated with memory improvement, then we would expect that cathodal tDCS of the right DLPFC specifically improves recognition memory as compared with cathodal tDCS of the left DLPFC and anodal tDCS.

## Experimental Investigation

### Participants

36 right-handed, native Italian speakers, participants (4 males, 32 females, age-range 21–29 years; education-range 13–16 years), were recruited from students’ population of University of Palermo. All participants were in good health and had no previous history of neurological or psychiatric illness. Written informed consent was obtained from all participants prior to participating in the study. The research protocol with tDCS was approved by the Institutional Review Board of the IRCCS Fondazione Santa Lucia, Rome, Italy. The experiments have been conducted according to the principles expressed in the Declaration of Helsinki.

### Materials

The materials used have been employed in previous studies where they have been described in detail [24]. The experimental task is an Italian version [37] of the Recognition Memory Test [38–39].

The stimuli used were unknown faces. In order to apply different stimuli in baseline and post tDCS sessions, we used two parallel forms of stimuli: the first (Form A) was the Faces Recognition Test from Smirni et al. [37]; the second (Form B) was a parallel form of the same test.

The faces were black and white photographs of Caucasian women, approximately 25 years old, with Italian physiognomic characteristics, neutral expression, and no obvious distinguishing features.

### Procedure

Procedures were identical in both forms. Stimuli were 30 and the tasks were computerized.

In the study phase, stimuli were presented individually in the center of a 15” computer screen over a white background for 2000 ms. The stimuli were preceded by a fixation point lasting 500 ms. The inter-stimulus interval (ISI) was 3500 ms.

Participants were instructed to judge whether the stimulus presented in the study phase was “pleasant” or “unpleasant”. This judgment task has been previously used [23, 40–41] to focus subjects’ attention in stimulus encoding. Participants responded by pressing one of two designated keys on the keyboard.

The recognition phase was administered after a delay interval of 20 minutes.

In the recognition phase, a three alternative forced choice recognition memory task was administered. Thirty stimulus triplets were presented. In each triplet, the target was presented with two other similar distractors, vertically arranged.

The target was presented in a balanced order either in the upper, lower or middle quadrant of the screen.

The distractors were two faces with physiognomic characteristics similar to the target. This similarity was previously established in a pilot study, in which participants were asked to judge the face similarity on the basis of hair and color configuration, eyes color and shape, nose and mouth shape.

The recognition trial began with a fixation point of 500 ms followed by the presentation of the triplets (target and two distractors) for 2000 ms. The ISI was 3500 ms.

Subjects were asked to recognize the previously presented stimuli by pressing one of three designated keys on the keyboard using the right index finger. If unsure they were asked to guess.

Responses were measured in terms of accuracy and reaction times (RTs). Accuracy was considered as the number of correct targets that participants were able to identify in the three forced-choice recognition memory test. The RTs were considered as the time interval from the onset of the test stimuli to the subject’s response.

### Transcranial Direct Current Stimulation (tDCS)

In a first experiment, involving 20 subjects (mean age: 23.56 ± 2.25 years; mean education: 14.7 ± 1.54 years), each subject underwent one session of sham tDCS and one session of left or right cathodal tDCS.

In a second experiment, involving 16 subjects (mean age: 24.7 ± 2.19 years); mean education: 15.5 ± 1.3 years), each subject underwent one session of sham tDCS and one session of left or right anodal tDCS.

Subjects were tested individually in a double daily session that lasted approximately 30 minutes per each session. Sham and real tDCS were separated by a 6 hours delay, a sufficient time to allow for the effects of tDCS to vanish out in the case of real preceding sham tDCS [42]. The order of the sessions within each experiment was randomized. Sham or real tDCS was applied during the 20’ delay interval between the study phase and the recognition phase.

tDCS was applied using a battery-driven BrainStim stimulator (EMS, Italy) with a pair of surface-soaked sponge electrodes (5 cm x 7 cm).

Cathodal tDCS was applied positioning the cathode over the right or left DLPFC (F3/F4 sites according to extended International 10–20 System for EEG electrode placement) and the anode above the contralateral shoulder. Anodal tDCS was applied positioning the anode over the right or left DLPFC site and the cathode above the contralateral shoulder. In both experiments, a constant current of 1mA intensity was applied on for 20 min.

For sham stimulation, the stimulator was turned off after 30 seconds of stimulation as previously described [43].

Accuracy (i.e. number of correctly recognized items) and RTs were analysed using ANOVA for repeated measures, with Side (left vs. right) as a between-subject effect, and Condition (sham vs. real tDCS) as a within-subject factor.

Two separate ANOVAs were conducted for cathodal and anodal tDCS.

## Results

All participants tolerated the stimulation well and did not complain of pain or discomfort during the stimulation. During sham stimulation, they did not realize that in one session they were stimulated only for the first 30 seconds, as verified with explicit questioning at the end of the last session.

### Experiment 1. Cathodal tDCS over the right/left DLPFC

#### Accuracy

**Fig 1:**
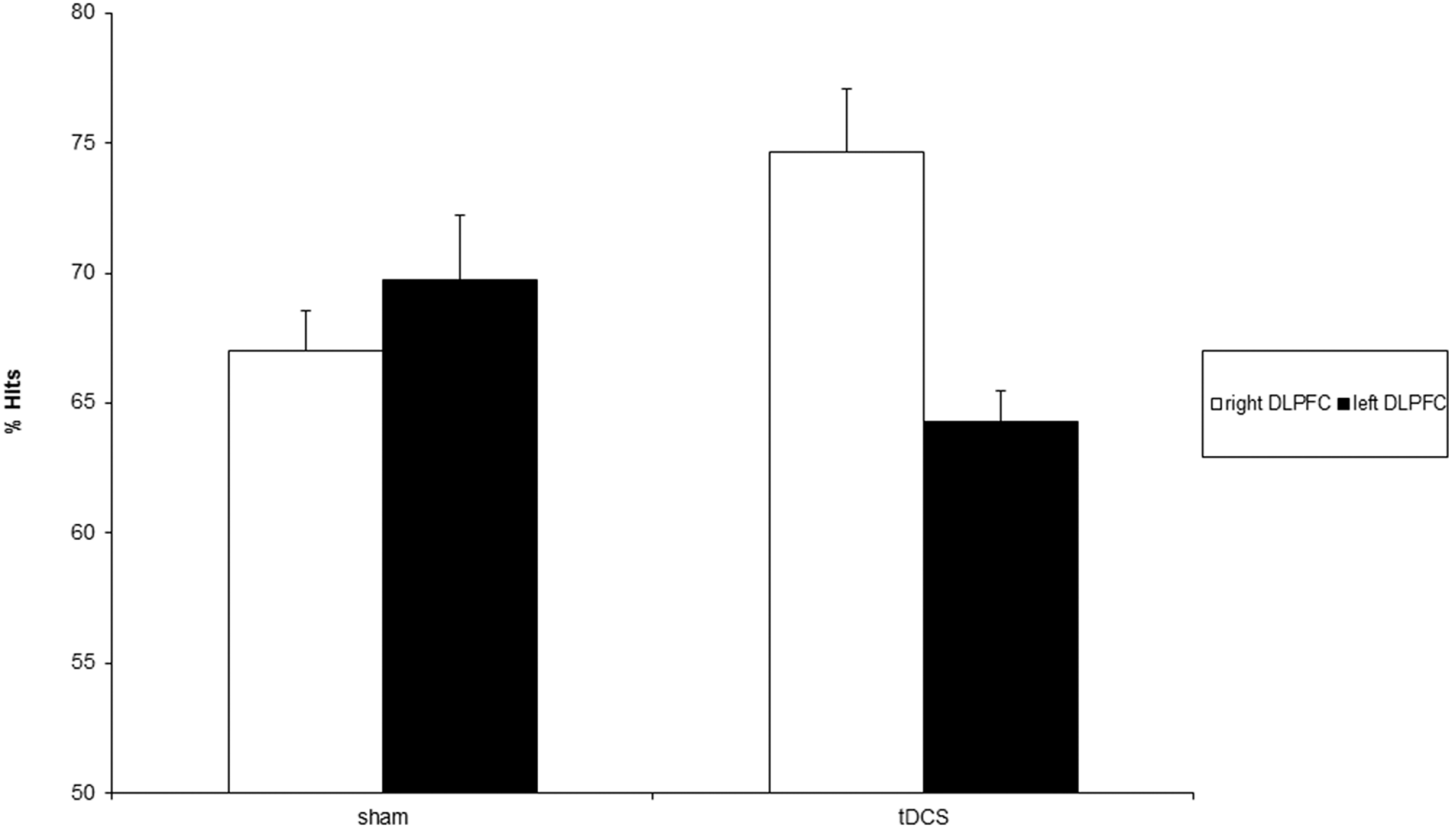
percentage of hits following sham and real cathodal tDCS of the right (white bars) and left (black bars) DLPFC. Error bars represent 1 SE of mean.

Side (F = 3.47; d.f. = 1,18; p>.08) and Condition (F = .31; d.f. = 1,18; p>.58) effects were not significant. The Side x Condition interaction was significant (F = 11.57; d.f. = 1,18; p < .01) (Fig 1).

Specifically, right cathodal tDCS significantly improved subjects’ accuracy when compared with sham tDCS (F = 7.84; d.f. = 1,18; p < .01). Furthermore, the effects of right cathodal tDCS significantly differed from those of left cathodal tDCS (F = 14.54; d.f. = 1,18; p < .01).

The two sham conditions did not differ between each other (F = .91; d.f. = 1,18; p>.35).

#### Reaction Times

Non significant effects of Side (F = .18; d.f. = 1,18; p>.68), Condition (F = .00; d.f. = 1,18; p>.95) and Side x Condition interaction (F = 1.87; d.f. = 1,18; p>.19) were found.

These results indicate that cathodal tDCS over the right DLPFC significantly improves non-verbal recognition memory performance, without any significant modulation of speed of response. Cathodal tDCS over the left DLPFC has no effect neither on accuracy nor on reaction times (Table 1).

**Table 1:** Average RTs in the different experimental conditions. Values in parenthesis represent SDs.

	Cathodal tDCS		Anodal tDCS	
	right DLPFC	left DLPFC	right DLPFC	left DLPFC
sham	1730.03 (255.5)	1734.1 (181.5)	1697.4 (273.7)	1755.2 (191.8)
real tDCS	1774.4 (230)	1693.3 (186.1)	1727 (234.4)	1729.5 (240.6)

### Experiment 2. Anodal tDCS over the right/left DLPFC

#### Accuracy

**Fig 2:**
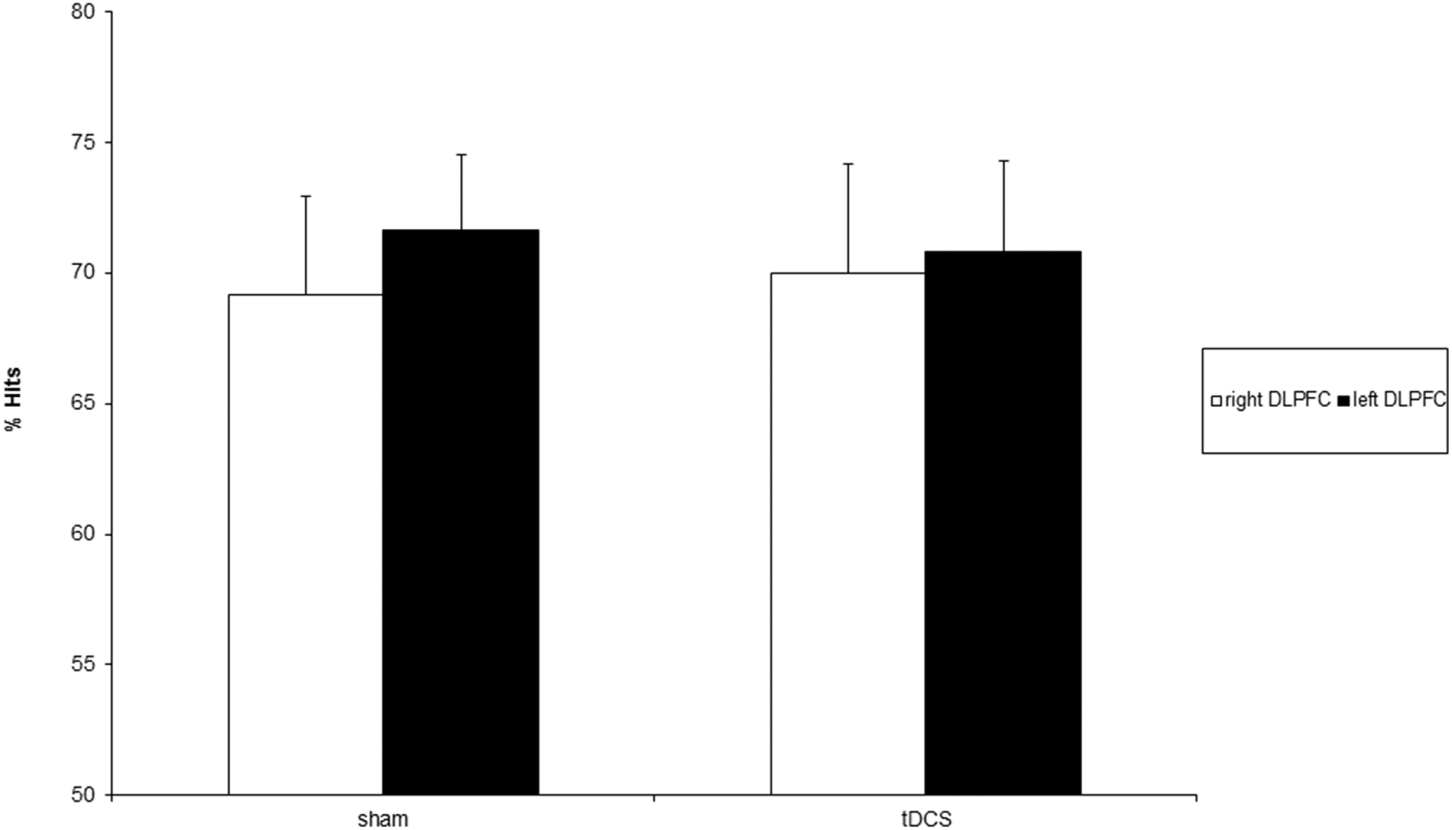
percentage of hits following sham and real anodal tDCS of the right (white bars) and left (black bars) DLPFC. Error bars represent 1 SE of mean.

Non significant main effects of Side (F = .19; d.f. = 1,14; p>.67), Condition (F = .00; d.f. = 1,14; p = 1) and Side x Condition interaction (F = .06; d.f. = 1,14; p>.81) were found (Fig 2).

#### Reaction Times

The Side (F = .16; d.f. = 1,14; p>.69] and Condition (F = 1.56; d.f. = 1,14; p>.23) effects, and the Side x Condition interaction (F = 1.44; d.f. = 1,14; p>.25) were not significant.

These results indicate that anodal tDCS does not has no effect neither on accuracy nor on reaction times in non-verbal recognition memory performance (Table 1).

## Discussion

The aim of the present study was to investigate the effects of cathodal and anodal tDCS over the left and the right DLPFC before retrieval in a non-verbal recognition memory task. The main results show that tDCS of the right DLPFC significantly modulates non-verbal recognition memory and its effects are hemisphere and polarity-dependent. In fact, cathodal tDCS of the right DLPFC improves accuracy in recognition memory without any significant modulation of speed response. Instead, cathodal tDCS of the left DLPFC has no effects in recognition memory performance, either on accuracy or on speed of response. Anodal tDCS over the right or left DLPFC has no effects in recognition memory performance accuracy or on speed of response.

In a previous study it was shown that rTMS inhibitory trains of the right DLPFC improved recognition memory performance in healthy controls for both verbal and non verbal stimuli, while left rTMS-inhibition had no effect in the recognition memory [23]. Similarly to the results obtained in healthy controls, the same study reported that rTMS-inhibition of the right DLPFC also improved recognition memory in Mild Cognitive Impairment patients with memory deficits. Also in this pathological model, inhibitory rTMS on the left DLPFC had no effect on recognition memory.

In the majority of published studies on the effects of prefrontal tDCS on long term memory, anodal tDCS of the left DLPFC was employed to improve memory performance, both in healthy subjects [7–9, 14–16, 44–45] and in patients with neurological disease [11–13, 46].

On the other hand, studies reporting memory facilitations following cathodal tDCS are more sparse. Kamida et al. [47] showed that cathodal tDCS in immature rats with status epilepticus-induced hippocampal cell loss prevented subsequent cognitive impairment. However, the authors interpreted this finding as closely related to neuromodulation of epilepsy rather than as an effect of cathodal tDCS per se.

Pisoni et al. [48] reported that bilateral stimulation of parietal and temporal cortices with anodal tDCS over the left hemisphere and cathodal tDCS over the right one boosted recognition memory performance,

In the light of prior studies showing improved recognition memory performance following 1 Hz rTMS trains of the right DLPFC, we interpret our findings as correlated to inhibition of the function of the right DLPFC. Indeed, there are increasing reports outlining the role of the right DLPFC in episodic memory [45,49], and correlating it to the role played by this brain area in inhibitory control [50–51] and in suppression of unwanted interfering memories [52]. According to this interpretation, one could speculate that inhibition of the right DLPFC by cathodal stimulation could have released the excitability of functionally interconnected areas of the medial temporal lobe, thus increasing subsequent recognition memory. In fact, since the depth of stimulation of TMS or tDCS is limited to a few centimeters, these techniques cannot directly stimulate the hippocampal and parahippocampal regions directly involved in episodic memory formation. However, stimulation of the DLPFC can propagate to distal brain structures [53–54], and it can also modify prefrontal-hippocampal connectivity [55]. If the prefrontal-hippocampal connection is mainly subserved by inhibitory fibers, its inhibition by cathodal tDCS (or by 1 Hz rTMS in previous studies) could likely result in hippocampal disinhibition during the recognition memory task. An alternative account posits that low-frequency rTMS or cathodal tDCS could act by increasing, rather than decreasing, the functional interaction of DLPFC and hippocampus. Related to this, Bilek et al. [55] showed that high-frequency rTMS, whose neural and behavioural effects are opposite as compared with those of low frequency rTMS, reduces prefrontal-hippocampal interaction.

Obviously, due to the limited spatial resolution of tDCS, one cannot rule out that tDCS influenced the excitability of other brain areas (such as the ventral prefrontal cortex) involved in the inhibitory network subserving memory retrieval. Future studies, combining brain stimulation with neuroimaging, could further explore the dynamic brain interactions between anterior and posterior brain areas involved in memory processes.

Consistently with the inhibitory account, recent work suggests that memory maintenance and consolidation depends upon the dynamic interaction between large-scale networks and the effective formation of memory traces is associated with suppressing interference from competing networks [56–58] (see also [59] for a discussion of the neural correlates involved in post-encoding processing).

Since tDCS was applied before retrieval, its effects could have modulated these consolidation processes.

Another, not mutually exclusive, explanation of the current results refers to transcallosal modulation of cortical excitability induced by brain stimulation techniques [60–61]. Indeed, it has repeatedly been demonstrated in healthy subjects as well as in patients with neurological disorders that inhibition of one hemisphere results in disinihibition of the contralateral one. Therefore, cathodal tDCS of the right DLPFC could have released from transcallosal inhibition the homologous region of the left hemisphere, resulting in increased excitability of the left DLPFC (see also [48] for a study reporting increased recognition memory following simultaneous right cathodal and left anodal tDCS). Modulation of the excitability of the left DLPFC could therefore be associated with the increased memory function, a result also consistent with the large literature previously discussed and reporting ameliorating effects on memory following anodal tDCS of the left DLPFC. The lack of significant effects on recognition memory of anodal tDCS of the left DLPFC in the present study could be explained with the different thresholds of cathodal vs. anodal tDCS. In fact, it has been shown that the intensity of the stimulation is a critical parameter for obtaining inhibitory vs. excitatory effects. For example, Boggio et al. [62] showed that 2 mA anodal tDCS over the left DLPFC significantly improved working memory accuracy in patients with Parkinson’s disease, whereas 1 mA anodal tDCS did not show any significant behavioral effect. This can also be the case with our finding on anodal stimulation of the left DLPFC, in which we observed only a non-significant trend toward better memory recognition. Possibly, higher electrical current intensity could significantly improve the recognition accuracy following left anodal prefrontal tDCS, an effect that is currently under investigation in our research group.

## References

[1] Ball K, Berch DB, Helmers KF, Jobe JB, Leveck MD, Marsiske M, et al Effects of cognitive training interventions with older adults: a randomized controlled trial. JAMA. 2002 11 13;288(18):2271–81. 12425704 10.1001/jama.288.18.2271 PMC2916176

[2] Willis SL, Tennstedt SL, Marsiske M, Ball K, Elias J, Koepke KM, et al Long-term effects of cognitive training on everyday functional outcomes in older adults. JAMA. 2006 12 20;296(23):2805–14. 17179457 10.1001/jama.296.23.2805 PMC2910591

[3] Acevedo A, Loewenstein DA. Nonpharmacological cognitive interventions in aging and dementia. J Geriatr Psychiatry Neurol. 2007 12;20(4):239–49. Review 18004010 10.1177/0891988707308808

[4] Boggio PS, Fregni F, Valasek C, Ellwood S, Chi R, Gallate J, et al Temporal lobe cortical electrical stimulation during the encoding and retrieval phase reduces false memories. PLoS One. 2009; 4(3):e4959 10.1371/journal.pone.0004959 19319182 PMC2655647

[5] Chi RP, Fregni F, Snyder AW. Visual memory improved by non-invasive brain stimulation. Brain Res. 2010 9 24;1353:168–75 10.1016/j.brainres.2010.07.062 20682299

[6] Penolazzi B, Di Domenico A, Marzoli D, Mammarella N, Fairfield B, Franciotti R, Brancucci A, Tommasi L. Effects of Transcranial Direct Current Stimulation on episodic memory related to emotional visual stimuli. PLoS One. 2010 5 13;5(5):e10623 10.1371/journal.pone.0010623 20498700 PMC2869343

[7] Javadi AH, Cheng P. Transcranial direct current stimulation (tDCS) enhances reconsolidation of long-term memory. Brain Stimul. 2013 7;6(4):668–74. 10.1016/j.brs.2012.10.007 23137702

[8] Javadi AH, Walsh V. Transcranial direct current stimulation (tDCS) of the left dorsolateral prefrontal cortex modulates declarative memory. Brain Stimul. 2012 7;5(3):231–41. 10.1016/j.brs.2011.06.007 21840287

[9] Javadi AH, Cheng P, Walsh V. Short duration transcranial direct current stimulation (tDCS) modulates verbal memory. Brain Stimul. 2012 10;5(4):468–74. 10.1016/j.brs.2011.08.003 21962975

[10] Jacobson TK, Howe MD, Schmidt B, Hinman JR, Escabí MA, Markus EJ. Hippocampal theta, gamma, and theta-gamma coupling: effects of aging, environmental change, and cholinergic activation. J Neurophysiol. 2013 4;109(7):1852–65. 10.1152/jn.00409.2012 23303862 PMC4868371

[11] Ferrucci R, Mameli F, Guidi I, Mrakic-Sposta S, Vergari M, Marceglia S et al Transcranial direct current stimulation improves recognition memory in Alzheimer disease. Neurology. 2008 8 12;71(7):493–8. 10.1212/01.wnl.0000317060.43722.a3 18525028

[12] Boggio PS, Valasek CA, Campanhã C, Giglio AC, Baptista NI, Lapenta OM et al Non-invasive brain stimulation to assess and modulate neuroplasticity in Alzheimer's disease. Neuropsychol Rehabil. 2011 10;21(5):703–16. Review. 10.1080/09602011.2011.617943 21942868

[13] Boggio PS, Ferrucci R, Mameli F, Martins D, Martins O, Vergari M, et al Prolonged visual memory enhancement after direct current stimulation in Alzheimer's disease. Brain Stimul. 2012 7;5(3):223–30. 10.1016/j.brs.2011.06.006 21840288

[14] Hammer A, Mohammadi B, Schmicker M, Saliger S, Münte TF. Errorless and errorful learning modulated by transcranial direct current stimulation. BMC Neurosci. 2011 7 22;12:72 10.1186/1471-2202-12-72 21781298 PMC3154153

[15] Manenti R, Brambilla M, Petesi M, Ferrari C, Cotelli M.Enhancing verbal episodic memory in older and young subjects after non-invasive brain stimulation.Front Aging Neurosci. 2013 9 11;5:49 10.3389/fnagi.2013.00049 24062685 PMC3769624

[16] Zwissler B, Sperber C, Aigeldinger S, Schindler S, Kissler J, Plewnia C. Shaping memory accuracy by left prefrontal transcranial direct current stimulation. J Neurosci. 2014 3 12;34(11):4022–6. 10.1523/JNEUROSCI.5407-13.2014 24623779 PMC3951698

[17] Simons JS, Spiers HJ. Prefrontal and medial temporal lobe interactions in long-term memory. Nat Rev Neurosci. 2003 8;4(8):637–48. 12894239 10.1038/nrn1178

[18] Squire LR. Memory systems of the brain: a brief history and current perspective. Neurobiol Learn Mem. 2004 11;82(3):171–7. Review. 15464402 10.1016/j.nlm.2004.06.005

[19] Milner B, Corsi P, Leonard G. Frontal-lobe contribution to recency judgements. Neuropsychologia. 1991;29(6):601–18. 1944864 10.1016/0028-3932(91)90013-x

[20] Wagner AD, Desmond JE, Glover GH, Gabrieli JD. Prefrontal cortex and recognition memory. Functional-MRI evidence for context-dependent retrieval processes. Brain. 1998 10;121(Pt 10):1985–2002. 9798751 10.1093/brain/121.10.1985

[21] McDermott KB, Buckner RL, Petersen SE, Kelley WM, Sanders AL. Set- and code-specific activation in frontal cortex: an fMRI study of encoding and retrieval of faces and words. J Cogn Neurosci. 1999 11;11(6):631–40. 10601744 10.1162/089892999563698

[22] Turriziani P., Oliveri M., Salerno S., Costanzo F., Koch G., Caltagirone C. et al Recognition memory and prefrontal cortex: dissociating recollection and familiarity processes using rTMS. Behav. Neurol. 2008 19, 23–27. 18413912 10.1155/2008/568057 PMC5452441

[23] Turriziani P, Smirni D, Oliveri M, Semenza C, Cipolotti L. The role of the prefrontal cortex in familiarity and recollection processes during verbal and non-verbal recognition memory: an rTMS study. Neuroimage. 2010 8 1;52(1):348–57. 10.1016/j.neuroimage.2010.04.007 20399276

[24] Turriziani P, Smirni D, Zappalà G, Mangano GR, Oliveri M, Cipolotti L. Enhancing memory performance with rTMS in healthy subjects and individuals with Mild Cognitive Impairment: the role of the right dorsolateral prefrontal cortex. Front Hum Neurosci. 2012 4 10;6:62 10.3389/fnhum.2012.00062 22514525 PMC3322484

[25] Kirchhoff BA, Wagner AD, Maril A, Stern CE. Prefrontal-temporal circuitry for episodic encoding and subsequent memory. J Neurosci. 2000 8 15;20(16):6173–80. 10934267 10.1523/JNEUROSCI.20-16-06173.2000 PMC6772579

[26] Buckner RL, Wheeler ME. The cognitive neuroscience of remembering. Nat Rev Neurosci. 2001 9;2(9):624–34. Review. 11533730 10.1038/35090048

[27] Fletcher PC, Henson RN. Frontal lobes and human memory: insights from functional neuroimaging. Brain. 2001 5;124(Pt 5):849–81. Review 11335690 10.1093/brain/124.5.849

[28] Rossi S, Cappa SF, Babiloni C, Pasqualetti P, Miniussi C, Carducci F, Babiloni F, Rossini PM. Prefrontal cortex in long-term memory: an "interference" approach using magnetic stimulation. Nat Neurosci. 2001 9;4(9):948–52. Erratum in: Nat Neurosci 2002 Oct;5(10):1017. 11528428 10.1038/nn0901-948

[29] Sandrini M, Cappa SF, Rossi S, Rossini PM, Miniussi C. The role of prefrontal cortex in verbal episodic memory: rTMS evidence. J Cogn Neurosci. 2003 8 15;15(6):855–61. 14511538 10.1162/089892903322370771

[30] Floel A, Poeppel D, Buffalo EA, Braun A, Wu CW, Seo HJ, Stefan K, Knecht S, Cohen LG. Prefrontal cortex asymmetry for memory encoding of words and abstract shapes. Cereb Cortex. 2004 4;14(4):404–9. 15028644 10.1093/cercor/bhh002

[31] Tulving E, Kapur S, Craik FI, Moscovitch M, Houle S. Hemispheric encoding/retrieval asymmetry in episodic memory: positron emission tomography findings. Proc Natl Acad Sci U S A. 1994 3 15;91(6):2016–20. Review. 8134342 10.1073/pnas.91.6.2016 PMC43300

[32] Habib R, Nyberg L, Tulving E. Hemispheric asymmetries of memory: the HERA model revisited. Trends Cogn Sci. 2003 6;7(6):241–245. 12804689 10.1016/s1364-6613(03)00110-4

[33] Spaniol J, Davidson PS, Kim AS, Han H, Moscovitch M, Grady CL. Event-related fMRI studies of episodic encoding and retrieval: meta-analyses using activation likelihood estimation. Neuropsychologia. 2009 7;47(8–9):1765–79. 10.1016/j.neuropsychologia.2009.02.028 19428409

[34] Aron AR, Robbins TW, Poldrack RA. Inhibition and the right inferior frontal cortex. Trends Cogn Sci. 2004 4;8(4):170–7. Review. 15050513 10.1016/j.tics.2004.02.010

[35] Tomita H, Ohbayashi M, Nakahara K, Hasegawa I, Miyashita Y. Top-down signal from prefrontal cortex in executive control of memory retrieval. Nature. 1999 10 14;401(6754):699–703. 10537108 10.1038/44372

[36] Juan CH, Muggleton NG. Brain stimulation and inhibitory control. Brain Stimul. 2012 4;5(2):63–9. 10.1016/j.brs.2012.03.012 Epub 2012 Apr 3. 22494830

[37] Smirni D, Turriziani P, Oliveri M, Smirni P, Cipolotti L. Standardizzazione di tre nuovi test di memoria di riconoscimento verbale e non verbale: uno studio preliminare. G. I. P. 2010 1, 227–245.

[38] Warrington EK. Recognition Memory Test. 1984 Windsor, Berks: NFER-Nelson.

[39] Warrington EK. The Camden Memory Tests. 1996 Hove, UK: Psychology Press.

[40] Cipolotti L, Bird C, Good T, Macmanus D, Rudge P, Shallice T. Recollection and familiarity in dense hippocampal amnesia: a case study. Neuropsychologia 2006 39, 151–172. 10.1016/j.neuropsychologia.2005.05.014 16023686

[41] Cipolotti L, Husain M, Crinion J, Bird CM, Khan SS, Losseff N. et al The role of the thalamus in amnesia: a tractography, high-resolution MRI and neuropsychological study. Neuropsychologia 2008 46, 2745–2758. 10.1016/j.neuropsychologia.2008.05.009 18597798

[42] Nitsche MA, Paulus W. Sustained excitability elevations induced by transcranial DC motor cortex stimulation in humans. Neurology. 2001 11 27;57(10):1899–901. 11723286 10.1212/wnl.57.10.1899

[43] Gandiga PC, Hummel FC, Cohen LG. Transcranial DC stimulation (tDCS): a tool for double-blind sham-controlled clinical studies in brain stimulation. Clin Neurophysiol. 2006 4;117(4):845–50. 16427357 10.1016/j.clinph.2005.12.003

[44] Kincses TZ, Antal A, Nitsche MA, Bartfai O, Paulus W. Facilitation of probabilistic classification learning by transcranial direct current stimulation of the prefrontal cortex in the human. Neuropsychologia. 2004; 42(1):113–117. 14615081 10.1016/s0028-3932(03)00124-6

[45] Manenti R, Cotelli M, Robertson IH, Miniussi C. Transcranial brain stimulation studies of episodic memory in young adults, elderly adults and individuals with memory dysfunction: a review. Brain Stimul. 2012 4;5(2):103–9. Review 10.1016/j.brs.2012.03.004 22503472

[46] Cotelli M, Manenti R, Brambilla M, Petesi M, Rosini S, Ferrari C et al Anodal tDCS during face-name associations memory training in Alzheimer's patients. Front Aging Neurosci. 2014 3 19;6:38 10.3389/fnagi.2014.00038 24678298 PMC3958642

[47] Kamida T, Kong S, Eshima N, Abe T, Fujiki M, Kobayashi H. Transcranial direct current stimulation decreases convulsions and spatial memory deficits following pilocarpine-induced status epilepticus in immature rats. Behav Brain Res. 2011 2 2;217(1):99–103 10.1016/j.bbr.2010.08.050 20826186

[48] Pisoni A, Turi Z, Raithel A, Ambrus GG, Alekseichuk I, Schacht A, Paulus W, Antal A. Separating recognition processes of declarative memory via anodal tDCS: boosting old item recognition by temporal and new item detection by parietal stimulation. PLoS One. 2015 3 27;10(3). 10.1371/journal.pone.0123085 PMC4376742 25816233

[49] Sandrini M, Censor N, Mishoe J, Cohen LG. Causal role of prefrontal cortex in strengthening of episodic memories through reconsolidation. Curr Biol. 2013 11 4;23(21):2181–4 10.1016/j.cub.2013.08.045 24206845 PMC3824257

[50] Knoch D, Gianotti LR, Pascual-Leone A, Treyer V, Regard M, Hohmann M, Brugger P. Disruption of right prefrontal cortex by low-frequency repetitive transcranial magnetic stimulation induces risk-taking behavior. J Neurosci. 2006 6 14;26(24):6469–72. 16775134 10.1523/JNEUROSCI.0804-06.2006 PMC6674035

[51] De Neys W, Vartanian O, Goel V.Smarter than we think: when our brains detect that we are biased. Psychol Sci. 2008 5;19(5):483–9. 10.1111/j.1467-9280.2008.02113.x 18466410

[52] Penolazzi B, Stramaccia DF, Braga M, Mondini S, Galfano G.Human memory retrieval and inhibitory control in the brain: beyond correlational evidence. J Neurosci. 2014 5 7;34(19):6606–10. 10.1523/JNEUROSCI.0349-14.2014 24806685 PMC6608142

[53] Bestmann S, Baudewig J, Siebner HR, Rothwell JC, Frahm J. Subthreshold high-frequency TMS of human primary motor cortex modulates interconnected frontal motor areas as detected by interleaved fMRI-TMS. Neuroimage. 2003 11;20(3):1685–96. 14642478 10.1016/j.neuroimage.2003.07.028

[54] Rounis E, Stephan KE, Lee L, Siebner HR, Pesenti A, Friston KJ et al Acute changes in frontoparietal activity after repetitive transcranial magnetic stimulation over the dorsolateral prefrontal cortex in a cued reaction time task. J Neurosci. 2006 9 20;26(38):9629–38. 16988033 10.1523/JNEUROSCI.2657-06.2006 PMC6674444

[55] Bilek E, Schäfer A, Ochs E, Esslinger C, Zangl M, Plichta MM, et al Application of high-frequency repetitive transcranial magnetic stimulation to the DLPFC alters human prefrontal-hippocampal functional interaction. J Neurosci. 2013 4 17;33(16):7050–6. 10.1523/JNEUROSCI.3081-12.2013 23595762 PMC6618883

[56] Kelly A, Uddin LQ, Biswal BB, Castellanos FX, and Milham M. Competition between functional brain networks mediates behavioral variability. Neuroimage 2008; 39: 527–537. 17919929 10.1016/j.neuroimage.2007.08.008

[57] Wermke M, Sorg C, Wohlschläger AM, Drzezga A. A new integrative model of cerebral activation, deactivation and default mode function in Alzheimer's disease. Eur J Nucl Med Mol Imaging. 2008 3;35 Suppl 1:S12 10.1007/s00259-007-0698-5 18299829

[58] Jacobs HI, Dillen KN, Risius O, Göreci Y, Onur OA, Fink GR, Kukolja J. Consolidation in older adults depends upon competion between resting-state networks. Front Aging Neurosci. 2015 1 9;6:344 10.3389/fnagi.2014.00344 25620930 PMC4288239

[59] van Kesteren MT, Fernández G, Norris DG, Hermans EJ. Persistent schema-dependent hippocampal-neocortical connectivity during memory encoding and postencoding rest in humans. Proc Natl Acad Sci U S A. 2010 4 20;107(16):7550–5. 10.1073/pnas.0914892107 20363957 PMC2867741

[60] Williams JA, Pascual-Leone A, Fregni F. Interhemispheric modulation induced by cortical stimulation and motor training. Phys Ther. 2010 3;90(3):398–410. 10.2522/ptj.20090075 20110339

[61] Oliveri M, Rossini PM, Traversa R, Cicinelli P, Filippi MM, Pasqualetti P, et al Left frontal transcranial magnetic stimulation reduces contralesional extinction in patients with unilateral right brain damage. Brain. 1999 9;122(Pt 9):1731–9. 10468512 10.1093/brain/122.9.1731

[62] Boggio PS, Ferrucci R, Rigonatti SP, Covre P, Nitsche M, Pascual-Leone A, et al Effects of transcranial direct current stimulation on working memory in patients with Parkinson's disease. J Neurol Sci. 2006 11 1;249(1):31–8. 16843494 10.1016/j.jns.2006.05.062

